# Correlates of mental disorder and harmful substance use in an indigenous Australian urban sample: an analysis of data from the Queensland Urban Indigenous Mental Health Survey

**DOI:** 10.1007/s00127-024-02648-8

**Published:** 2024-03-20

**Authors:** Tabinda Basit, Maree Toombs, Damian Santomauro, Harvey Whiteford, Alize Ferrari

**Affiliations:** 1 Queensland Centre for Mental Health Research, The Park Centre for Mental Health Treatment, Level 3 Dawson House, Wacol, QLD 4076 Australia; 2 School of Public Health, The University of Queensland, Herston, QLD Australia; 3 Institute for Health Metrics and Evaluation, University of Washington, Seattle, USA; 4 School of Public Health, University of Sydney, Sydney, NSW Australia

**Keywords:** Epidemiology, Psychiatry, Social determinants, Mental health, Indigenous health

## Abstract

**Purpose:**

Limited data exists on the relationship between sociodemographic and cultural variables and the prevalence of specific mental and substance use disorders (MSDs) among Indigenous Australians, using diagnostic prevalence data. This paper utilises data from the Queensland Urban Indigenous Mental Health Survey (QUIMHS), a population-level diagnostic mental health survey, to identify socioeconomic and cultural correlates of psychological distress and specific MSDs in an urban Indigenous Australian sample.

**Methods:**

Using a mixture of household sampling (door-knocking) and snowball sampling (promotion of the survey in the community), 406 participants aged 18 to 89 were recruited across key locations in Southeast Queensland. The study investigated various demographic, socioeconomic, and cultural factors as predictors of psychological distress (measured by the Kessler-5) and MSD diagnoses (utilising the Composite International Diagnostic Interview, CIDI 3.0) using a series of univariate logistic regressions.

**Results:**

Individuals in unstable housing (homeless, sleeping rough) and those reporting financial distress were more likely to experience an MSD in the past 12 months and throughout their lifetime. Individuals reporting lower levels of connection and belonging, limited participation in cultural events, and lower empowerment were more likely to have a lifetime mental disorder.

**Conclusion:**

This data emphasises the importance of addressing systemic and social determinants of health when designing and delivering community mental health services and underscores the need for holistic approaches when working with Indigenous communities.

**Supplementary Information:**

The online version contains supplementary material available at 10.1007/s00127-024-02648-8.

## Introduction

The prevalence of mental disorders and substance use disorders (MSDs) in Aboriginal and Torres Strait Islander peoples of Australia (henceforth respectfully referred to as Indigenous Australians) is a complex and multifaceted issue that is influenced by many social, cultural, and historical factors [1], [2]. There is evidence to suggest that Indigenous Australians face elevated levels of psychological distress, are more susceptible to MSDs, and are more likely to access community mental health services compared to the general population [3–5]. These health disparities are commonly accepted as stemming from traditional socioeconomic factors that affect the general population, as well as compounding cultural and historical factors that are specific to Indigenous Australian communities [6–8]. These relate to ongoing impacts of colonisation, which manifest in ongoing experiences of subjugation, marginalization, discrimination, and disrupted connections to identity, health, and overall well-being [7, 9].

National data (2004-05 National Health Survey and the 2004-05 and 2012-13 National Aboriginal and Torres Strait Islander Health Survey), has found a significant association between elevated psychological distress and a range of sociodemographic variables in Indigenous Australians. Being female, socioeconomic factors (including lower education levels, unemployment, lower income, and overall area-level disadvantage in non-remote regions), and cultural factors (including experiences of racism and a history of family removal) were associated with experiencing high/very high levels of psychological distress [6, 8]. Similar associations with socioeconomic factors, education, disconnection from culture or community, and interpersonal violence have been found in First Nations populations of comparable OECD countries [10–14]. Importantly, the literature also presents evidence of the converse pattern. Studies have consistently shown that factors such as connection to community, language, and spirituality have predictive value for positive mental health outcomes [11]. In Australia and New Zealand, research has demonstrated that maintaining a strong connection to Indigenous culture and residing in areas with a high ethnic density serve as protective factors against mental disorders by fostering resilience and well-being. Even in the face of significant socioeconomic challenges, these factors have been found to mitigate psychological distress [15].

Outside of correlates of wellbeing and psychological distress, there are notably fewer research studies investigating correlates of MSDs (such as major depressive disorder (MDD), generalised anxiety disorder (GAD) or post-traumatic stress disorder (PTSD)) and their prevalence for Indigenous Australians. To date, two studies have explored the relationship between sociodemographic variables (including sex, age, location (urban, regional etc.), income, marital status, and accommodation) and MSD prevalence. Both studies found the only significant predictor of mental disorder to be residential location, but the direction of the findings were mixed. Urban locations were a protective factor against current major depressive disorder for older adults in one study [16], while the other found that residing in a remote/very remote location was protective against any MSD, any mood disorder, any anxiety disorder and any substance disorder across the lifetime [17]. Other research in Australia and New Zealand has demonstrated that maintaining a strong connection to Indigenous culture and residing in areas with a high ethnic density serve as protective factors against some MSDs by fostering resilience and well-being [15].

## Aim

The Queensland Urban Indigenous Mental Health Survey (QUIMHS) was conducted in 2022 and aimed to address the lack of representative population-based epidemiological data on MSDs in Indigenous Australian samples [18]. It undertook a co-design process with Indigenous Australian stakeholders to develop its survey methodology and adapt the instrumentation for use within an urban Indigenous sample in Southeast Queensland (SEQ). Findings identified that 46.5% of the sampled population had experienced a mental disorder or harmful substance use within the 12 months prior. The most prevalent disorders were major depressive episodes and post-traumatic stress disorder, at 24.6% and 19.9% of the sample, respectively. The study also found that 45.8% of the sample reported high or very high psychological distress [18]. This research paper will use the data from the QUIMHS data to identify and explore the socioeconomic and cultural correlates of psychological distress and specific MSDs respectively in an urban Indigenous Australian sample. Understanding the correlates of both psychological distress and MSDs can add to our understanding about whether these factors differ by the type of psychological presentation and better inform service planning and public health initiatives.

There are a few points of importance to acknowledge when seeking to add to the knowledge base. First, Indigenous Australians and those working within community already have a thorough understanding and lived experience of the factors associated with presentations of psychological distress and mental disorder. The intent of this paper is not to subvert this valuable source of data or to add ‘novel’ findings; rather, it is to support, supplement and corroborate what is already known using rigorous epidemiological methods. Second, although it may be argued that identifying the correlates of specific common mental disorders and psychological distress within Indigenous Australians is reinforcing a deficits narrative, it also holds potential to inform service planners and providers of factors that promote health and wellbeing and may be of use when designing public health and health promotion initiatives.

## Methods

### Data source

Data collection for the QUIMHS survey was conducted between February and October of 2022. Ethics approval for the QUIMHS Survey was provided by the Townsville Health Services HREC (HREC/2020/QTHS/61,158) and was ratified by UQ HREC. Study methods were co-designed with Indigenous Australian stakeholders and community participants and are reported in full elsewhere [18]. Seven trained Indigenous Australian interviewers administered structured face to face or video interviews with participants. Survey participants were recruited using a mixed-methods sampling strategy, comprised of randomised household sampling and snowball convenience sampling across key locations in SEQ. The final sample consisted of 406 Indigenous Australians aged between 18 and 89 years.

### Survey Instrument

The QUIMHS survey instrument included: (a) A measure of psychological distress in the past 30 days, using the Kessler Psychological Distress Scale adapted for use with Indigenous Australian peoples (Kessler-5) [8], (b) a standardised diagnostic instrument, the Composite International Diagnostic Interview (CIDI 3.0) [19], that produced estimates of mental disorder prevalence and severity, specifically, major depressive disorder, generalised anxiety disorder, and post-traumatic stress disorder in the past 12 months and lifetime (see Table 1), and (c) a custom substance use screening module developed in reference to the CIDI 3.0, the AUDIT [20] and the Severity of Dependence Scale [21] that collected information on harmful substance use and indications of probable dependence (see supplementary information, Table 6 for a list of included disorders). Although the CIDI 3.0 is not yet validated for use in Indigenous Australian populations, it is used widely in population surveys nationally, globally and has been used in an Indigenous Queensland population in custody [22]. As such it best allowed for comparison of prevalence data. The QUIMHS pilot study specifically focused on assessing the appropriateness and accuracy of the CIDI 3.0 for use with Indigenous Australians, and those methods and results are reported in more detail elsewhere [18, 23]. The QUIMHS instrument also included some qualitative response options to allow participants to respond verbatim when the standardised response options did not adequately reflect their experience. The four items relevant to this research paper are detailed in the supplementary information, Table 7. More information about the QUIMHS survey instrument and research methods are described in detail elsewhere [18].

### Sociodemographic and cultural factors

The QUIMHS survey instrument gathered a range of data on participants’ demographic, socioeconomic and cultural factors. The variables of interest, their definitions, and response options are listed below in Table 1.

**Table 1:** Sociodemographic and cultural factors included in the QUIMHS survey

Variable	Definition	Response options
Age	Age of participant	Continuous variable (18–99)
Sex	Sex of participant	Male, female, other
Indigenous status	Participant’s Indigenous identification	Aboriginal, Aboriginal and Torres Strait Islander, Torres Strait Islander
Marital status	Participant’s marital status	Divorced/separated, widowed, married/de facto, partnered, single, other
Highest level of schooling	Participant’s highest level of schooling	Finished age 12–15 years, finished aged 16–17 years, finished school
Highest level of tertiary education	Participant’s highest level of secondary education	Bachelor, postgraduate degree, certificate, diploma, associate degree, no qualifications
Employment status	Participant’s employment status	Paid employment, government payments, unemployed, studying
Living situation	Participant’s living situation	Homeowner, renting, staying with friend or family, sleeping rough, homeless, other
Financial stress (in last 12 months)	Questions about financial stress, including: • In the past 12 months, has your household always been able to pay for electricity, petrol, or telephone bills on time? • Gone without meals, or been unable to afford groceries? • Sought assistance from welfare or community organisations (e.g., the Salvation Army)? • Sought financial help from friends or family (i.e., to pay for food, rent, bills, etc.)?	Yes, no
History of incarceration	Participant’s history of incarceration	Yes as a youth, yes as an adult, yes as a youth and an adult, no
Identification of Mob	Participant’s mob(s) or tribe(s)	Name of mob(s) (open text), don’t know/unsure
Identification of Country	If participant considers a place to be Country	Yes, no, not sure
Time on country	Questions about the time since participant was last on country, including: • Do you currently live on country? • Have you ever spent time/lived on country before? • How long has it been since you were last there?	Yes, no, days/months/years
History of family removal	Questions about history of family removals, including: • Were either of your parents or grandparents’ part of the Stolen Generations? • Were you a part of the Stolen Generations?	Yes (one or both parents), yes (one or both grandparents), yes (self), No
Questions on cultural experiences	Questions about cultural experiences, including: 1. I am proud to identify myself to others as an Aboriginal and/or Torres Strait Islander Person, 2. I feel a sense of connection and belonging to my Aboriginal and/or Torres Strait Islander culture, 3. I participate in Aboriginal and/or Torres Strait Islander community events and activities (e.g., National Aborigines and Islanders Day Observance Committee (NAIDOC), Sorry Business), and 4. I feel empowered and strong to make positive choices for myself, my family and my community, 5. I experience racism/discrimination because of my Aboriginal and/or Torres Strait Islander status.	Never, rarely, sometimes, often, always. Scored on a Likert scale: 5, 4, 3, 2, 1 respectively.
“Cultural connection” composite score	The cultural experience items on cultural pride, connection, participation and empowerment were (items 1–4) were summed to create a total “cultural connection” score.	Continuous variable from 4–16, where lower scores represent higher levels of pride, connection, participation and empowerment.

### Analysis

Analyses were performed using R software, version 4.2.2 [24]. A series of univariate logistic regressions investigated associations between selected sociodemographic and cultural variables (independent variables), with (1) measures of high/very high psychological distress and (2) 12-month and lifetime mental disorder diagnoses and harmful substance use (dependent variables). The svyglm function within the survey package was used to accommodate for the design of the survey [25]. Data were weighted by location (Local Government Areas), age, and sex using the distribution of Indigenous Australians within each respective age-sex-location group reported in the 2021 Australian Census. To facilitate data exploration and improve sample sizes, some variable response options were aggregated into broader levels. For example, the item: “*I am proud to identify myself to others as an Aboriginal and/or Torres Strait Islander Person”*, had five response options aggregated into two categories of never/rarely/sometimes vs. often/always. Where variables encompassed multiple response categories, a reference group was designated for comparison; on most occasions, this was the most normative or common response option. The regression analyses produced odds ratios (OR), interpreted as the likelihood of having either elevated psychological distress or a mental disorder or harmful substance use across various levels of a given sociodemographic variable. T-tests were conducted to explore the relationship between the “cultural connection” composite score and those who did/did not meet criteria for a mental disorder or harmful substance use. Analyses were applied universally across both sex and all age groups, primarily due to constraints in sample size and statistical power, maximising our ability to detect statistically significant effects across age and sex. All outputs were reported with 95% confidence intervals (CI) and p-values where applicable.

Qualitative comments were selected to display alongside the quantitative results to add participant perspectives and richness to the data where possible. This was done in a supplemental manner as there were insufficient comments relating to the sociodemographic and cultural variables of interest to warrant a more comprehensive qualitative analysis. A summary of the qualitative responses relevant to this paper can be found in the supplementary information, Table 7.

## Results

### Sample characteristics

The total sample consisted of 406 participants, with 72.2% females and 27.3% male. The average age of participants was 42.1 years. Most participants self-identified as Aboriginal (92.4%), with some identifying as both Aboriginal and Torres Strait Islander (5.2%), and 2.5% identified solely as Torres Strait Islander. Table 2 presents the total sample counts, accompanied by unweighted percentages, categorised by various socio-demographic characteristics. More information about the QUIMHS sample is detailed elsewhere [18].

**Table 2:** Sample socio-demographic characteristics

Variable	Variable levels	% of total sample	Sample Count^a^
Age (years)	18 to 29	23.6	96
	30 to 39	22.4	91
	40 to 49	21.2	86
	50 to 59	19.2	78
	60 +	13.5	55
Sex^b^	Female	72.0	293
	Male	27.3	111
Indigenous status	Aboriginal	92.4	375
	Aboriginal and Torres Strait Islander	5.2	21
	Torres Strait Islander	2.5	10
Marital status	Divorced/Separated/Widowed	7.1	29
	Married/De facto/Partnered	55.7	226
	Single/Other	37.2	151
Highest level of schooling^b^	Finished aged 12 to 15 years	9.6	39
	Finished aged 16 to 17 years	5.2	21
	Finished school	84.2	342
Highest level of tertiary education^b^	Bachelor / Postgraduate degree	18.7	76
	Certificate/Diploma/Associate degree	46.6	189
	No qualifications / Prefer not to say	34.7	141
Employment status^b^	Paid employment	63.1	256
	Government payments	22.9	93
	Unemployed	10.8	44
	Studying	4.2	17
Living situation	Homeowner	29.1	118
	Renting	57.9	235
	Staying with friend or family	10.8	44
	Sleeping rough/Homeless/Other	2.2	9

^a^Unweighted counts provided

^b^Due to very small cell counts, some response options to these variables have been suppressed

### Correlates of psychological distress

#### Sociodemographic correlates

Correlates of psychological distress are presented in Table 3. Participants experiencing financial distress were between 3 and 4 times more likely to experience high or very high psychological distress in the past 30 days (as measured by the Kessler-5). This included those who were unable to pay their bills (OR = 3.1, [95% CI: 1.9–5.2]), unable to afford groceries (OR = 4.4, [95% CI: 2.4–7.8]), and had to seek welfare payments (OR = 3.0, [95% CI: 1.8–4.9]). In qualitative comments, some participants identified “*financial issues”* and “*finances”* as precipitators of poor psychological health, but also barriers to treatment. Those who were unemployed were over twice as likely to experience high or very high psychological distress in the past 30 days (OR = 2.2, [95% CI: 1.2–4.1]). Trends suggested that those with without tertiary qualifications (OR = 1.3, [95% CI: 0.8-2.0]), those sleeping rough (OR = 3.4, [95% CI: 0.6–18.1]), and those who had a history of incarceration (OR = 1.3, [95% CI: 0.6–3.2]) were likely to experience more psychological distress, however these did not reach significance.

#### Cultural correlates

Those who did not consider a certain place to be their Country or were unsure of their Country were 2 times more likely to have experienced high or very high psychological distress in the past 30 days (OR = 2.0, [95% CI: 1.1–3.5]). When asked about strategies to cope with mental health, some participants mentioned country: *“Cultural connection to country is the best way,”* and “*There are a lot of young people out there that don’t have connection to country or know about the importance or the help it can do for you.”* Trends also suggested that those who did not know who their mob is were more likely to experience psychological distress. Participants that reported they sometimes, rarely or never felt a sense of connection and belonging (OR = 3.4, [95% CI: 2.0–6.0]), participated in community events (OR = 2.1, [95% CI: 1.3–3.4]), and felt empowered and strong to make positive choices (OR = 4.8, [95% CI: 2.4–9.6]), were up to 4 times more likely to have experienced high or very high psychological distress. In qualitative comments, some participants indicated the role of community when seeking help for their mental health, such as *“talking to other people in community,”* and *“I attend a men’s group… he’s an Islander fella and he brings in culture.”*

**Table 3:** Sociodemographic and cultural correlates of psychological distress

Socio-demographic variable	Odds Ratio	95% CI*	N
Age (years)			
18–39	1.2	(0.8–1.8)	187
40+ (Ref)			219
Marital status			
Married/De facto/Partnered (Ref)			226
Divorced/Separated/Widowed	0.9	(0.4–2.2)	29
Single/Other	1.2	(0.8–1.9)	151
Highest year of school completed			
Year 12/equivalent (Ref)			342
Did not complete school	1.1	(0.6–1.9)	64
Highest tertiary qualification			
Certificate/Diploma/Associate degree (Ref)			189
Bachelor/Post-graduate degree	0.8	(0.4–1.4)	76
No qualifications/Prefer not to say	1.3	(0.8–2.0)	141
Employment status			
Paid employment (Ref)			256
Government payments	1.6	(0.9–2.7)	87
Unemployed/Studying without source of income	2.2*	**(1.2–4.1)**	63
Living situation			
Renting (Ref)			235
Homeowner	0.7	(0.5–1.2)	118
Staying with friends/family	0.9	(0.4–1.7)	44
Sleeping rough/Homeless/Other	3.4	(0.6–18.1)	9
Financial stress (in last 12 months)			
Able to pay bills (Ref)			309
Unable to pay bills	3.1*	**(1.9–5.2)**	97
Able to afford groceries (Ref)			328
Unable to afford groceries	4.4*	**(2.4–7.8)**	78
Did not seek assistance from welfare (Ref)			297
Sought assistance from welfare	3.0*	**(1.8–4.9)**	109
Did not seek financial health from friends/family (Ref)			266
Sought financial help from friends/family	1.5	(0.5–4.3)	140
History of incarceration			
No incarceration (Ref)			380
Incarceration (youth and/or adult)	1.3	(0.6–3.2)	26
**Cultural variable**	**Odds Ratio**	95% CI*	**N**
Identification of Mob			
Mob identified (Ref)			363
No Mob identified – don’t know/unsure	1.7	(0.9–3.5)	43
Identification of Country			
Considers a place to be Country (Ref)			338
Does not consider a place to be Country/not sure	2.0*	**(1.1–3.5)**	68
Living on country			
Does not currently live on country (Ref)			332
Currently living on country	1.3	(0.8–2.2)	74
Has never lived on country (Ref)			300
Has lived on country (Ref)	0.7	(0.5–1.2)	106
History of family removals			
Not part of Stolen Generations (Ref)			395
Part of Stolen Generations	0.7	(0.2–2.5)	11
Parents were not part of Stolen Generations (Ref)			364
Parents were part of Stolen Generations	1.4	(0.7–2.8)	42
Grandparents were not part of Stolen Generations (Ref)			247
Grandparents were part of Stolen Generations	0.9	(0.6–1.4)	159
Cultural experiences			
Always/often proud to identify (Ref)			394
Sometimes/rarely/never proud to identify	1.5	(0.5–5.2)	12
Always/often feel a sense of connection and belonging (Ref)			328
Sometimes/rarely/never feel a sense of connection and belonging	3.4*	**(2.0–6.0)**	78
Always/often participates in community events (Ref)			297
Sometimes/rarely/never participates in community events	2.1*	**(1.3–3.4)**	109
Always/often feels empowered and strong to make positive choices (Ref)			353
Sometimes/rarely/never feels empowered and strong to make positive choices	4.8*	**(2.4–9.6)**	53
Sometimes experiences racism/discrimination (Ref)			177
Always/often experiences racism/discrimination	0.9	(0.6–1.4)	134
Rarely/never experiences racism/discrimination	1.0	(0.6–1.8)	95

*Reference groups are signified as (Ref)*

* Significance based on 95% Confidence Intervals (CI)

The composite “cultural connection” score was also correlated with K5 scores, where higher scores represented higher levels of psychological distress. There was a moderate correlation (*r* = 0.369, *p* < 0.05) between the two variables, indicating that lower levels of cultural connection were associated with higher levels of psychological distress.

### Correlates of mental disorders and harmful substance use in the last 12 months

#### Sociodemographic correlates

The 12-month correlate results are presented in Table 4 for mental disorders and harmful substance use aggregates and at the disorder level in the supplementary information (Table 8). Single people were 2.4 times as likely to experience harmful substance use than those married, in de facto or partnered relationships (OR = 2.4 [CI: 1.2–5.1]), with a similar trend for those divorced, separated or widowed. Those indicating their current living situation as sleeping rough or experiencing homelessness were 5.5 times more likely to have a mental disorder or harmful substance use (OR = 5.5 [CI: 1.1–27.7]), compared with individuals who were renting. Those experiencing some form of financial stress during the previous year were 2 times as likely to have experienced any mental disorder or harmful substance use in the same period, which included, those unable to pay bills (OR = 2.0 [CI: 1.2–3.3]), afford groceries (OR = 2.0 [CI: 1.2–3.4]), seeking assistance from welfare (OR = 2.1 [CI: 1.2–3.3]), or financial assistance from family (OR = 2.1 [CI: 1.3–3.3]). At the disorder level, patterns largely followed trends presented for the aggregate group (see supplementary information, Table 8). Notably, respondents who were unemployed were almost 2 times as likely to have a major depressive episode in the last 12 months (OR = 1.9 [CI: 1.0-3.6] and those on government payments were 7 times more likely to have indications of probable illicit drug dependence (OR = 7.3 [CI: 1.3–41.8]) compared to those in paid employment. Individuals who had a history of incarceration, either as youth or adults, were almost 5 times more likely to have any harmful substance use (OR = 4.8 [CI: 1.8–12.7]) and probable illicit drug dependence (OR = 31.6 [CI: 6.7-148.2]) in the past 12 months compared to those without a history of incarceration. Some participant comments on precipitators of poor psychological health reflected these findings. One participant stated: *“Prices in real estate and everything else has gone up so much, I can’t afford anything or find anywhere to live… [there’s a] possibility of losing my home and kids.”* Another participant reported: *“I became homeless, separated from my partner… I was couch surfing and sleeping in my car.”* One comment stated: “*Wrongful incarceration.”* Results looking at tertiary education were mixed, with those with a professional degree associated with increased risk of any mental disorder (OR = 1.8 [CI: 1.0-3.1]) relative to those with technical degrees, but decreased risk of any harmful substance use (OR = 0.2 [CI: 0.0-0.9]). Those without tertiary qualifications seemed to be less likely to experience mental disorder or harmful substance use, but this did not reach significance. No statistically significant differences in the prevalence of disorders among various age groups or completion of high school were found.

#### Cultural correlates

Participants who responded sometimes, rarely or never to experiences of connection (OR = 1.7 [CI: 1.0-2.9]), community event participation (OR = 1.8 [CI: 1.1–2.8]), and feelings of empowerment (OR = 2.0 [CI: 1.1–3.7]) were approximately 2 times more likely to have any mental disorder when compared to those who responded often or always to the same item. See supplementary information, Table 8 for how this varied at the disorder level. No statistically significant associations were found amongst experiences of racism, identification of Mob, Country, or history of family removals.

**Table 4:** Sociodemographic and cultural correlates of any mental disorder and harmful substance use in the last 12 months

	Any mental disorder or harmful substance use	Any mental disorder	Any harmful substance use	
Sociodemographic variable	Odds Ratio (95% CI)			N
Age				
40+				
18–39	1.1 (0.7–1.7)	1.1 (0.7–1.6)	1.3 (0.7–2.7)	187
Marital status				
Married/De facto/Partnered (Ref)				226
Divorced/Separated/Widowed	1.1 (0.5–2.7)	1.1 (0.5–2.6)	2.0 (0.5–7.4)	29
Single/Other^a^	1.5 (1.0-2.4)	1.4 (0.9–2.1)	2.4 (1.2–5.1)*	151
Highest year of school completed				
Year 12/equivalent (Ref)				342
Did not complete school^b^	1.2 (0.7–2.1)	0.9 (0.5–1.7)	1.6 (0.6-4.0)	64
Highest tertiary qualification				
Certificate/Diploma/Associate degree (Ref)				189
Bachelor/Post-graduate degree	1.3 (0.8–2.3)	1.8 (1.0-3.1)*	0.2 (0.0-0.9)*	76
No qualifications/Prefer not to say	0.7 (0.5–1.2)	0.8 (0.5–1.3)	0.5 (0.2–1.2)	141
Employment status				
Paid employment (Ref)				256
Government payments	1.5 (0.9–2.5)	1.3 (0.7–2.2)	1.7 (0.7–4.1)	87
Unemployed/Studying without source of income	1.3 (0.7–2.4)	1.4 (0.8–2.5)	1.1 (0.4–3.1)	63
Living situation				
Renting (Ref)				235
Homeowner	0.9 (0.6–1.5)	1.0 (0.6–1.6)	0.7 (0.3–1.7)	118
Staying with friends/family	0.8 (0.4–1.6)	1.1 (0.5–2.1)	0.7 (0.2–2.4)	44
Sleeping rough/Homeless/ Other	5.5 (1.1–27.7)*	1.6 (0.4–6.6)	12.3 (3.0-49.7)*	9
Financial stress (in last 12 months)				
Able to pay bills (Ref)				309
Unable to pay bills	2.0 (1.2–3.3)*	1.9 (1.2–3.1)*	2.0 (0.9-4.0)	97
Able to afford groceries (Ref)				328
Unable to afford groceries	2.0 (1.2–3.4)*	2.0 (1.2–3.3)*	1.6 (0.7–3.6)	78
Did not seek assistance from welfare (Ref)				297
Sought assistance from welfare	2.1 (1.3–3.3)*	1.7 (1.1–2.7)*	1.9 (0.9–3.9)	109
Did not seek financial health from friends/family (Ref)				266
Sought financial help from friends/family	2.0 (1.3–3.2)*	1.9 (1.2–2.9)*	1.9 (0.9–3.7)	140
History of incarceration				
No incarceration (Ref)				380
Incarceration (youth and/or adult)	1.3 (0.6–3.2)	0.8 (0.3-2.0)	4.8 (1.8–12.7)*	26
	Any mental disorder or harmful substance use	Any mental disorder	Any harmful substance use	
Cultural variable	Odds Ratio (95% CI)			N
Identification of Mob				
Mob identified (Ref)				363
No Mob identified – don’t know/unsure	1.4 (0.7–2.8)	1.1 (0.6–2.2)	1.2 (0.4–2.4)	43
Identification of Country				
Considers a place to be Country (Ref)				338
Does not consider a place to be Country/not sure	1.0 (0.6–1.8)	1.1 (0.6–1.9)	1.3 (0.5–3.1)	68
Living on country				
Does not currently live on country (Ref)				332
Currently living on country	1.0 (0.6–1.8)	1.0 (0.6–1.7)	0.9 (0.4–2.4)	74
Has never lived on country (Ref)				300
Has lived on country	1.5 (0.9–2.5)	1.4 (0.8–2.3)	1.0 (0.4–2.2)	106
History of family removals				
Not part of Stolen Generations (Ref)				395
Part of Stolen Generations	1.4 (0.4–5.1)	0.8 (0.2–2.8)	3.2 (0.6–18.4)	11
Parents were not part of Stolen Generations (Ref)				364
Parents were part of Stolen Generations	1.1 (0.6–2.2)	0.9 (0.5–1.8)	2.1 (0.8–5.3)	42
Grandparents were not part of Stolen Generations (Ref)				247
Grandparents were part of Stolen Generations	0.8 (0.5–1.3)	0.8 (0.5–1.3)	1.0 (0.5-2.0)	159
Cultural experiences				
Always/often proud to identify (Ref)				394
Sometimes/rarely/never proud to identify	0.9 (0.3-3.0)	1.1 (0.3–3.7)	0.6 (0.1–5.2)	12
Always/often feel a sense of connection and belonging (Ref)				328
Sometimes/rarely/never feel a sense of connection and belonging	1.5 (0.9–2.5)	1.7 (1.0-2.9)*	0.8 (0.3–2.1)	78
Always/often participates in community events (Ref)				297
Sometimes/rarely/never participates in community events	1.5 (1.0-2.4)	1.8 (1.1–2.8)*	0.7 (0.3–1.6)	109
Always/often feels empowered and strong to make positive choices (Ref)				353
Sometimes/rarely/never feels empowered and strong to make positive choices	1.9 (1.0-3.6)*	2.0 (1.1–3.7)*	1.0 (0.4–2.8)	53
Sometimes experiences racism/discrimination (Ref)				177
Always/often experiences racism/discrimination	0.8 (0.5–1.3)	0.7 (0.4–1.1)	1.6 (0.7–3.4)	134
Rarely/never experiences racism/discrimination	0.7 (0.4–1.1)	0.7 (0.4–1.2)	0.5 (0.1–1.5)	95

*Reference groups are signified as (Ref)*

* Significance based on 95% Confidence Intervals (CI)

### Correlates of mental disorders across the lifetime

As with 12-month correlates, participants experiencing financial stress, including being unable to pay bills (OR = 1.9 [CI: 1.1–3.2]), afford groceries (OR = 2.0 [CI: 1.1–3.5]), or seeking financial help from friends/family (OR = 2.0 [CI: 1.3–2.3]) were at increased risk of any mental disorder. Participants who were single were more likely to experience PTSD in the lifetime (OR = 1.7 [CI: 1.1–2.7]). Participants reporting lower levels of connection (OR = 2.3 [CI: 1.3–4.1]), community participation (OR = 2.4 [CI: 1.4–4.1]) and feelings of empowerment (OR = 2.1 [CI: 1.1–4.2]) were also at increased risk of any mental disorder. These results varied by disorder (see supplementary information, Table 9, for all lifetime correlates). Trends indicated those who could not identify who their Mob were more likely to experience all mental disorders; this reached significance for PTSD (OR = 2.2 [CI: 1.1–4.2]). Participants currently living on country were less likely to have experienced GAD in the lifetime (OR = 0.3 [CI: 0.1–0.7]), but there was no observable trend for other disorders. Items on racism and history of family removals showed inconclusive or mixed results.

When looking at the composite “cultural connection” score and its relationship with mental disorders, t-tests demonstrated that those with mental disorders (MDE, GAD and PTSD) had a higher mean cultural connection score (indicating lower levels of pride, connection, participation and empowerment) than those who did not have that disorder (see Table 5). The relationship between harmful substance use and the composite cultural connection scores was not significant.

**Table 5:** Composite cultural connection score and mental disorder

Comparison	N	Mean	CI (95%)	t	(df)	p
Mental disorders (12 month)						
Any mental disorder	178	7.0	6.6–7.3	3.79	404	< 0.01*
Without any mental disorder	227	6.0	5.7–6.3			
Major depressive episode	38	6.8	6.3–7.3	1.90	404	0.05*
Without major depressive episode	363	6.3	6.0-6.5			
Generalised anxiety disorder	31	7.5	6.6–8.3	2.63	404	< 0.01*
Without generalised anxiety disorder	375	6.3	6.1–6.6			
Post-traumatic stress disorder	77	6.9	6.4–7.4	1.99	404	< 0.05*
Without post-traumatic stress disorder	329	6.3	6.0-6.6			
Any harmful substance use	158	6.0	5.2–6.8	-0.99	399	0.32
Without any harmful substance use	248	6.5	6.2–6.7			
Mental disorders (lifetime)						
Any mental disorder	242	6.9	6.6–7.2	5.31	404	< 0.01*
Without any mental disorder	164	5.6	5.3-6.0			
Major depressive episode	176	6.8	6.5–7.2	3.19	404	< 0.01*
Without major depressive episode	230	6.1	3.7–6.4			
Generalised anxiety disorder	67	7.1	6.5–7.2	2.69	404	< 0.01*
Without generalised anxiety disorder	339	6.3	6.0-6.5			
Post-traumatic stress disorder	135	7.0	6.6–7.4	3.78	404	< 0.01*
Without post-traumatic stress disorder	271	6.1	5.8–6.4			

## Discussion

This research is among the first of its kind to examine sociodemographic and cultural correlates of diagnostic data on mental disorder and harmful substance use amongst an Aboriginal and Torres Strait Islander sample using a population level survey. Various socio-demographic and cultural factors showed significant correlations with a higher prevalence of psychological distress, mental disorders or harmful substance use. For instance, individuals who were experiencing homelessness or rough sleeping were over five times more likely to have experienced a mental disorder or harmful substance use in the past year when compared to those renting. Those who reported financial stress were approximately twice as likely to have encountered a mental disorder or harmful substance use in the last 12 months compared to individuals who reported no financial stress. It’s important to note that the relationship between these variables and mental health can be intricate and bidirectional. For example, although the measures of financial stress were based on experiences in the past 12 months, they were also related to increased rates of lifetime mental disorder. Poorer mental health status across the lifetime would impact on people’s ability to engage in activities that secure economic stability. For many individuals grappling with a mental disorder or harmful substance use, the attainment and maintenance of stable employment and housing can be formidable challenges. This, in turn, exacerbates their physical and mental well-being, economic stability, access to social support networks, and utilisation of healthcare services [26].

Due to limited variability in response types and small sample sizes, the logistic regression output was at times challenging to interpret. Very few associations between items on Mob, Country and history of family removals and mental disorder or harmful substance use were found. Previous research has found contrary results in terms of the effects of living on Country or within Aboriginal communities [16]. Broadly speaking, it may be challenging to separate the potential protective social and cultural elements of being on country from area level disadvantage [6]. Additionally, depending on the individual, connection to country may be experienced as a connection to the land, family, culture, or spirit, and this may vary from person to person. As a result of insufficient power in this study, we were unable to identify an overarching relationship between participants’ history of family removals and mental disorder or harmful substance use, however removal is known to be detrimental to the mental health and wellbeing of Indigenous peoples [27]. Due to the long history of government policy on forced removals [27], the impact on an individuals’ mental health may vary depending on the individual circumstance, historical timeframe and intervention they or their family members were subject to. Using broad, single-item measures of whether a person is currently living on Country or their personal and familial history of forced removals may miss the necessary nuance required to appropriately explore this relationship.

Importantly, connection to culture as measured by this survey and its relationship to rates of mental disorder was a key finding. Participants reporting lower levels of connection and belonging to their culture, limited participation in cultural events and activities, and feelings of disempowerment were at least twice as likely to have experienced a lifetime mental disorder compared to those indicating stronger cultural connection and belonging, greater participation in cultural activities, and a stronger sense of empowerment. These findings underscore the protective role of cultural identity in the health and well-being of Indigenous Australians [28]. Further research aimed at quantifying this impact is crucial, especially within the context of mental disorder prevention and intervention at a population level.

## Limitations

This paper was exploratory in nature and focused on providing descriptive associations to inform policy makers, public health service providers or planners, rather than the development of a predictive model. As a result, we did not correct our significance tests for multiple comparisons or for Type 1 error. While this preserves our ability to describe practical associations that may be useful to this audience, it does raise the probability that one or more of our significant findings may be due to chance. The research examined whether those experiencing racism had differing prevalence of mental disorder. The analyses produced inconclusive results, with those always/often experiencing racism having similar odds-ratios to those rarely/never experiencing racism. When we investigated the distribution of responses, a large number of participants (177/407) endorsed the “sometimes” response option, and most cases of mental disorder fell within this group. This exploration suggests that additional levels are needed in these response options, as limited categorical response options such as “sometimes” may capture a variety of participant experiences. Some qualitative comments indicated experiences of racism and discrimination as immediately preceding a mental health episode, with one participant identifying *“racism and discrimination at work,”* and another commenting: *“toxic energy from the workplace… bullying and racism.”* The scope of the QUIMHS research limited the extent and depth to which culturally derived experiences could be explored. The establishment of more comprehensive measures of cultural connection, racism, and discrimination, and further exploration of how these may impact mental disorder is required.

QUIMHS, a cross-sectional survey of Indigenous Australian adults in SEQ, represents the largest diagnostic survey of its kind conducted in this region [18]. The study employed research methods that included cultural adaptations, frameworks, and measures shaped by close collaboration with Indigenous stakeholders and community members in SEQ. This collaborative approach is a notable strength of the research. However, it’s important to acknowledge several limitations.

QUIMHS’ sample size limited statistical power in our analyses to detect all significant effects, and results should be interpreted with caution. Females were overrepresented in this sample. Although analyses were weighted to adjust for this, participation rates in mental health surveys as well as access to mental health services often see higher engagement and participation rates from females [4, [29]. Ways to engage males, particularly younger cohorts, in mental health research initiatives are required. Some sociodemographic variable levels had very few endorsements (e.g., those reporting homelessness, a history of incarceration or being part of the stolen generation), which limits the power with which significant effects can be detected. Furthermore, our findings may not be directly transferable to other Indigenous Australian communities, particularly those residing in rural and remote areas. Due to the cross-sectional design of the survey, the observed relationships between prevalence and other variables signify associations rather than establishing causality. The survey combined random household door knocking with community engagement efforts [18]. This approach captured segments of the population not residing in traditional households, a group often missed by surveys relying solely on door knocking, however, may have introduced a self-selection bias. Participants more inclined to strongly identify with the survey’s subject matter, actively engage in their community, have access to healthcare services, or feel comfortable discussing their mental health may have been overrepresented in the sample.

## Conclusion/Future research

The QUIMHS survey represents a groundbreaking epidemiological study in Australia due to its scale, focusing on mental disorder prevalence, harmful substance use, and service utilisation within the wider Indigenous Australian community in SEQ. The survey’s findings point to high rates of mental disorders and harmful substance use, but importantly, this paper speaks to the substantial impact of secure housing, stable financial circumstances, and cultural connectedness as potential protective factors against the same. This data underlines and strengthens what service providers, clinicians and community already know: the importance of addressing systemic and social determinants of health, particularly when planning for the design and delivery of mental health services targeted at community. More nuanced research looking at experiences of racism, the diversity of impacts of a history of forced removals and the protective effects of living on or being connected to country is required.

## Electronic supplementary material

Below is the link to the electronic supplementary material.

Supplementary Material 1

## Data Availability

The QUIMHS data that supports the findings of this study is held in line with the CARE Principles for Indigenous Data Governance, which refer to the right of Indigenous peoples to exercise ownership over Indigenous Data and ensure that it is used to support wellbeing, self-determination and justice-reinvestment. As such, the data are available from the authors upon reasonable request.

## References

[1] Kirmayer LJ, Brass G (2016) Addressing global health disparities among indigenous peoples. Lancet 388(10040):105–106. 10.1016/s0140-6736(16)30194-5 27108233 10.1016/S0140-6736(16)30194-5

[2] Cooke M, Mitrou F, Lawrence D, Guimond E, Beavon D (2007) Indigenous well-being in four countries: an application of the UNDP’s Human Development Index to Indigenous peoples in Australia, Canada, New Zealand, and the United States. BMC Int Health Hum Rights 7(1):9. 10.1186/1472-698x-7-9 18096029 10.1186/1472-698X-7-9 PMC2238768

[3] Australian Institute of Health and Welfare (2011) The Health and Welfare of Australia’s Aboriginal and Torres Strait Islander people: an overview. Australian Institute of Health and Welfare., Canberra. 10.25816/5ebcbd26fa7e4

[4] Australian Bureau of Statistics. National Aboriginal and Torres Strait Islander Health Survey (2018) -19 financial year. Canberra: Australian Bureau of Statistics.: Australian Bureau of Statistics; 2019. [Available from: https://www.abs.gov.au/statistics/people/aboriginal-and-torres-strait-islander-peoples/national-aboriginal-and-torres-strait-islander-health-survey/latest-release].

[5] Jorm AF, Bourchier SJ, Cvetkovski S, Stewart G (2012) Mental health of indigenous australians: a review of findings from community surveys. Med J Aust 196(2):118–121. 10.5694/mja11.10041 22304605 10.5694/mja11.10041

[6] Cunningham J, Paradies YC (2012) Socio-demographic factors and psychological distress in indigenous and non-indigenous Australian adults aged 18–64 years: analysis of national survey data. BMC Public Health 12:95. 10.1186/1471-2458-12-95 22296820 10.1186/1471-2458-12-95 PMC3340306

[7] King M, Smith A, Gracey M (2009) Indigenous health part 2: the underlying causes of the health gap. Lancet 374(9683):76–85. 10.1016/s0140-6736(09)60827-8 19577696 10.1016/S0140-6736(09)60827-8

[8] McNamara BJ, Banks E, Gubhaju L, Joshy G, Williamson A, Raphael B et al (2018) Factors relating to high psychological distress in indigenous australians and their contribution to indigenous-non-indigenous disparities. Aust N Z J Public Health 42(2):145–152. 10.1111/1753-6405.12766 29384250 10.1111/1753-6405.12766

[9] Thurber KA, Brinckley M-M, Jones R, Evans O, Nichols K, Priest N, et al. Population-level contribution of interpersonal discrimination to psychological distress among Australian Aboriginal and Torres Strait Islander adults, and to Indigenous–non-Indigenous inequities: cross-sectional analysis of a community-controlled First Nations cohort study. The Lancet. 2022; 400(10368): 2084-94. 10. 10.1016/S0140-6736(22)01639-7 10.1016/S0140-6736(22)01639-7 PMC9807286 36502846

[10] Australian Institute of Health and Welfare (2015) The health and welfare of Australia’s Aboriginal and Torres Strait Islander peoples: 2015. AIHW, Canberra. 10.25816/5ebcbd26fa7e4

[11] Nelson SE, Wilson K (2017) The mental health of indigenous peoples in Canada: a critical review of research. Soc Sci Med 176:93–112. 10.1016/j.socscimed.2017.01.021 28135694 10.1016/j.socscimed.2017.01.021

[12] Brave Heart MY, Lewis-Fernández R, Beals J, Hasin DS, Sugaya L, Wang S et al (2016) Psychiatric disorders and mental health treatment in American indians and Alaska natives: results of the national epidemiologic survey on Alcohol and related conditions. Soc Psychiatry Psychiatr Epidemiol 51(7):1033–1046. 10.1007/s00127-016-1225-4 27138948 10.1007/s00127-016-1225-4 PMC4947559

[13] W.D.C Commission on Civil Rights. A Quiet Crisis: Federal Funding and Unmet Needs in Indian Country. [Available from http://purl.access.gpo.gov/GPO/LPS49018 ]

[14] Baxter J, Kani Kingi T, Tapsell R, Durie M, Mcgee MA, Australian (2006) New Z J Psychiatry 40(10):914–923. 10.1080/j.1440-1614.2006.01911.x 10.1080/j.1440-1614.2006.01911.x 16959018

[15] Black E, Kisely S, Alichniewicz K, Toombs M (2017) Mood and anxiety disorders in Australia and New Zealand’s indigenous populations: a systematic review and meta-analysis. Psychiatry Res 255:128–138. 10.1016/j.psychres.2017.05.015 28544944 10.1016/j.psychres.2017.05.015

[16] Shen YT, Radford K, Daylight G, Cumming R, Broe TGA, Draper B (2018) Depression, suicidal behaviour, and mental disorders in older aboriginal australians. Int J Environ Res Public Health 15(3). 10.3390/ijerph15030447 10.3390/ijerph15030447 PMC5876992 29510527

[17] Nasir BF, Toombs MR, Srinivas K-C, Kisely S, Gill NS, Black E et al (2018) Common mental disorders among indigenous people living in regional, remote and metropolitan Australia: a cross-sectional study. BMJ Open 8(6). 10.1136/bmjopen-2017-020196 10.1136/bmjopen-2017-020196 PMC6042557 29961007

[18] Ferrari A, Basit T, Santomauro D, Chambers TK, Shadid J, Lindstom A et al (2023) The Staying Deadly Survey: The Queensland Urban Indigenous Mental Health Survey Report. 10.14264/1c4e5ce

[19] The World Health Organization World Mental Health Composite International Diagnostic Interview (WHO WMH-CIDI): World Health Organisation (2004) [Available from: https://www.hcp.med.harvard.edu/wmhcidi/index.php].

[20] The World Health Organization AUDIT: The Alcohol Use Disorders Identification Test, Guidelines for Use in Primary Care. Department of Mental Health and Substance Dependence (2001) [Available from: https://www.who.int/publications/i/item/WHO-MSD-MSB-01.6a

[21] Gossop M, Darke S, Griffiths P, Hando J, Powis B, Hall W et al (1995) The severity of dependence scale (SDS): psychometric properties of the SDS in English and Australian samples of heroin, cocaine and amphetamine users. Addiction 90(5):607–614. 10.1046/j.1360-0443.1995.9056072.x 7795497 10.1046/j.1360-0443.1995.9056072.x

[22] Heffernan EB, Andersen KC, Dev A, Kinner S (2012) Prevalence of mental illness among Aboriginal and Torres Strait Islander people in Queensland prisons. Med J Aust 197(1):37–41. 10.5694/mja11.11352 22762230 10.5694/mja11.11352

[23] Basit T, Anderson M, Lindstrom A, Santomauro DF, Whiteford HA, Ferrari AJ (2023) Diagnostic accuracy of the Composite International Diagnostic Interview (CIDI 3.0) in an urban indigenous Australian sample. Australian New Z J Psychiatry 57(2):283–290. 10.1177/00048674221150361 36688275 10.1177/00048674221150361

[24] R Core Team. R: a language and environment for statistical computing. Vienna: R Foundation for Statistical Computing (2019) Available from: [https://www.R-project.org/]

[25] Lumley T (2010) Complex surveys: a guide to analysis using. Wiley. 10.1002/9780470580066

[26] Mental health and experiences of homelessness: Australian Bureau of Statistics (2014) [Available from: https://www.abs.gov.au/statistics/health/mental-health/mental-health-and-experiences-homelessness/latest-release#cite-window1]

[27] Bringing them home: report of the National Inquiry into the Separation of Aboriginal and Torres Strait Islander Children from their Families. Sydney: Human Rights and Equal Opportunity Commission: Human Rights and Equal Opportunity Commission (1997) [Available from: https://humanrights.gov.au/our-work/bringing-them-home-report-1997]

[28] McIvor O, Napoleon A, Dickie K (2009) Language and Culture as protective factors for At-Risk communities. J Aboriginal Health 5:6–25. 10.18357/ijih51200912327

[29] Burgess PM, Pirkis JE, Slade TN, Johnston AK, Meadows GN, Gunn JM (2009) Service use for mental health problems: findings from the 2007 National Survey of Mental Health and Wellbeing. Aust N Z J Psychiatry 43(7):615–623. 10.1080/00048670902970858 19530018 10.1080/00048670902970858

[30] National Health and Medical Research Council, Ethical conduct in research with Aboriginal and Torres Strait Islander Peoples and communities: Guidelines for researchers and stakeholders.: Commonwealth of Australia: Canberra (2018) [Available from: https://www.nhmrc.gov.au/about-us/resources/ethical-conduct-research-aboriginal-and-torres-strait-islander-peoples-and-communities]

