# Supplementary material for: Correlates of mental disorder and harmful substance use in an indigenous Australian urban sample: an analysis of data from the Queensland Urban Indigenous Mental Health Survey
Source: Soc Psychiatry Psychiatr Epidemiol. 2024 Mar 20;60(1):201–13. doi: 10.1007/s00127-024-02648-8 (PMC11790685; doi:10.1007/s00127-024-02648-8)
Supplement: Supplementary file 1 — Supplementary Material 1 [file 127_2024_2648_MOESM1_ESM.docx]

## Supplementary Information

Table 6: Included disorders

| Included Disorders | Definition |
| --- | --- |
| Major depressive disorder (12-month and lifetime prevalence) | A mood disorder characterised by one or more major depressive episodes i.e., at least 2 weeks of depressed mood or loss of interest accompanied by at least four additional symptoms of depression that include changes in appetite or weight, decreased energy and feelings of worthlessness. |
| Dysthymia (12-month and lifetime prevalence) | A mood disorder characterised by chronic depression, demonstrating less severe but longer-lasting symptoms than major depressive disorder. Dysthymia involves the experience of chronically depressed mood for most of the day, more days than not, for at least two years. |
| Generalised anxiety disorder (12-month and lifetime prevalence) | An anxiety disorder characterised by persistent and excessive anxiety and worry, occurring more days than not for a period of at least 6 months. Feelings of anxiety and worry are accompanied by at least three additional symptoms from a list including restlessness, irritability and muscle tension. |
| Post-traumatic stress disorder (12-month and lifetime prevalence) | Characterised by the re-experiencing of an extremely traumatic event accompanied by symptoms of increased arousal and by avoidance of stimuli associated with the trauma. Symptoms must be present for more than 1 month and the disturbance must cause clinically significant distress or impairment in social, occupational or other important areas of functioning. |
| Harmful substance use (12-month prevalence) | Characterised by a pathological pattern of behaviours related to use of a substance that comprise of impaired control, social impairment, risky use, and pharmacological criteria (tolerance and withdrawal). The following substances may be included:   - Alcohol - Cannabis - Hallucinogens - Inhalants - Opioids - Sedatives - Stimulants |

Table 7: Summary of qualitative comments

| Survey Item | Response distribution | | Summary of relevant responses |
| --- | --- | --- | --- |
|  | **n** |  |  |
| Was there something going on in your life shortly before that episode started that caused it to occur?  Other: | 20 | Family, domestic or sexual violence |  |
|  | 13 | Interpersonal stressors |  |
|  | 7 | Housing event | All seven responses identified sudden displacement or housing instability as being precipitators to a mental health episode. |
|  | 6 | Child removal |  |
|  | 6 | Health event |  |
|  | 6 | Workplace stressors | Of the six responses, two referred to racism or discrimination at work as being precipitators to a mental health episode. The remaining responses referred to general workplace stress or events in the workplace. |
|  | 5 | Bereavement |  |
|  | 5 | Justice system event | One response referred to incarceration as a precipitator to a mental health episode. The remaining four spoke about issues with the police/court system. |
|  | 5 | Life transition |  |
|  | 2 | Drug use |  |
|  | 2 | Financial stressors | Both responses identified financial stress as being precipitators to a mental health episode. |
|  | 1 | Spiritual event | This response was not detailed enough to interpret. |
| Apart from the things just mentioned or seeking professional help, have you ever used any of these strategies to help cope with your mental health problems in the past 12 months?  Other: | 13 | Relaxation strategies |  |
|  | 8 | Self-care and development |  |
|  | 5 | Medication |  |
|  | 4 | Therapy |  |
|  | 2 | Support person or group |  |
|  | 2 | Pets |  |
|  | 2 | Faith based strategies |  |
|  | 2 | Cultural support | Both responses referred to cultural groups as being helpful. |
|  | 1 | Drug use |  |
|  | 1 | Online support |  |
| Considering your mental health care in the past 12 months, which of the following forms of help did you receive from (those/that) (hospitalisation(s)/consultation(s)?  Other: | 7 | Psychological skills development |  |
|  | 4 | Support to access services |  |
|  | 3 | Complementary or alternative therapies |  |
|  | 2 | Cultural interventions | Of the two responses, one spoke about connection to country, and one spoke about traditional healing practices as helpful and important. |
|  | 1 | Traditional medical interventions |  |
|  | 1 | Did not receive adequate help |  |
|  |  |  |  |
| Is there anything that we have not covered regarding your use of healthcare services, your preferences, or your access to healthcare services that you think is important to mention? | 50 | Service accessibility and awareness |  |
|  | 24 | Cultural safety and considerations | Of the 24 responses, two spoke about connection to country, and two spoke about traditional healing practices within community as helpful and important.  The remaining responses referred to Indigenous Australian staffing, lateral violence, and specific services. |
|  | 7 | Individual stories |  |
|  | 5 | Mental health stigma |  |
|  | 5 | Service cost | All five responses identified cost of services as a limiting or prohibitive factor to access. |

Table 8. Sociodemographic and cultural correlates of 12-month mental disorders at disorder level

|  | **Major depressive episode** | **Generalised anxiety disorder** | **Post-traumatic stress disorder** | **Probable alcohol dependence** | **Probable illicit drug dependence** |  |
| --- | --- | --- | --- | --- | --- | --- |
| **Sociodemographic variable** | **Odds Ratio (95% CI)** | | | | | **N** |
| Age |  |  |  |  |  |  |
| 40+ (Ref) |  |  |  |  |  | **219** |
| 18-39 | 0.8 (0.5-1.3) | 1.2 (0.6-2.5) | 1.5 (0.9-2.6) | 1.0 (0.4-2.2) | 1.7 (0.4-7.4) | 187 |
| Marital status |  |  |  |  |  |  |
| Married/De facto/Partnered (Ref) |  |  |  |  |  | 226 |
| Divorced/Separated/Widowed | 0.7 (0.3-2.1) | 1.2 (0.3-6.0) | 1.2 (0.4-2.5) | 3.1 (0.8-12.3) | - | 29 |
| Single/Other^a^ | 1.0 (0.6-1.7) | 1.5 (0.7-3.4) | 1.8 (1.0-3.1) | 2.0 (0.8-4.8) | - | 151 |
| Highest year of school completed |  |  |  |  |  |  |
| Year 12/equivalent (Ref) |  |  |  |  |  | 342 |
| Did not complete school^b^ | 0.7 (0.3) | 1.6 (0.6-4.1) | 0.9 (0.4-1.9) | 1.3 (0.4-4.2) | 4.5 (1.0-20.1) | 64 |
| Highest tertiary qualification |  |  |  |  |  |  |
| Certificate/Diploma/Associate degree (Ref) |  |  |  |  |  | 189 |
| Bachelor/Post-graduate degree | **2.1 (1.1-3.9)*** | 1.5 (0.6-4.0) | 0.8 (0.4-1.7) | - | - | 76 |
| No qualifications/Prefer not to say | 1.0 (0.6-1.7) | 0.9 (0.4-2.2) | 0.6 (0.3-1.1) | 0.4 (0.1-1.2) | 1.0 (0.2-4.4) | 141 |
| Employment status |  |  |  |  |  |  |
| Paid employment (Ref) |  |  |  |  |  | 256 |
| Government payments | 1.2 (0.6-2.2) | 1.5 (0.6-3.8) | 1.3 (0.7-2.4) | 1.1 (0.4-3.5) | **7.3 (1.3-41.8)*** | 87 |
| Unemployed/Studying without source of income | **1.9 (1.0-3.6)*** | 1.7 (0.7-4.5) | 0.8 (0.4-1.7) | 1.7 (0.5-5.0) | 3.8 (0.5-29.2) | 63 |
| Living situation |  |  |  |  |  |  |
| Renting (Ref) |  |  |  |  |  | 235 |
| Homeowner | 0.9 (0.5-1.5) | 1.1 (0.5-2.6) | 1.0 (0.5-1.8) | 1.2 (0.5-3.0) | - | 118 |
| Staying with friends/family | 0.6 (0.2-1.3) | 1.1 (0.3-3.8) | 1.2 (0.5-2.8) | - | 1.0 (0.1-9.3) | 44 |
| Sleeping rough/Homeless/ Other | 1.8 (0.4-7.1) | 3.4 (0.6-18.4) | 1.6 (0.3-8.0) | 6.6 (1.2-35.8) | **16.1 (2.6-101.0)*** | 9 |
| Financial stress (in last 12 months) |  |  |  |  |  |  |
| Able to pay bills (Ref) |  |  |  |  |  | 309 |
| Unable to pay bills | **1.8 (1.1-3.0)*** | **2.8 (1.3-6.0)*** | 1.5 (0.8-2.7) | 1.1 (0.4-2.9) | **9.8 (1.9-51.3)*** | 97 |
| Able to afford groceries (Ref) |  |  |  |  |  | 328 |
| Unable to afford groceries | **2.0 (1.2-3.5)*** | 2.1 (0.9-4.8) | 1.2 (0.6-2.3) | 0.7 (0.2-2.4) | **5.3(1.3-22.5)*** | 78 |
| Did not seek assistance from welfare (Ref) |  |  |  |  |  | 297 |
| Sought assistance from welfare | 1.6 (1.0-2.8) | 1.7 (0.8-3.7) | **1.9 (1.1-3.3)*** | 0.8 (0.3-2.1) | **7.7 (1.5-39.7)*** | 109 |
| Did not seek financial help from friends/family (Ref) |  |  |  |  |  | 266 |
| Sought financial help from friends/family | **1.9 (1.2-3.1)*** | 0.5 (0.2-1.3) | 1.6 (0.9-2.7) | 1.4 (0.6-3.4) | 2.0 (0.5-8.5) | 140 |
| History of incarceration |  |  |  |  |  |  |
| No incarceration (Ref) |  |  |  |  |  | 380 |
| Incarceration (youth and/or adult) | 0.7 (0.2-2.1) | 2.4 (0.7-7.6) | 1.2 (0.4-3.5) | 1.4 (0.3-5.6) | **31.6 (6.7-148.2)*** | 26 |
|  |  |  |  |  |  |  |
|  | **Major depressive episode** | **Generalised anxiety disorder** | **Post-traumatic stress disorder** | **Probable alcohol dependence** | **Probable illicit drug dependence** |  |
| **Cultural variable** | **Odds Ratio (95% CI)** | | | | | **N** |
| Identification of Mob |  |  |  |  |  | **­** |
| Mob identified (Ref) |  |  |  |  |  | 363 |
| No Mob identified – don’t know/unsure | 1.0 (0.5-2.1) | 2.2 (0.8-6.0) | 1.4 (0.6-3.0) | 0.6 (0.1-2.7) | 0.9 (0.1-7.2) | 43 |
| Identification of Country |  |  |  |  |  |  |
| Considers a place to be Country (Ref) |  |  |  |  |  | 338 |
| Does not consider a place to be Country/not sure | 1.2 (0.6-2.1) | 0.3 (0.1-1.2) | 1.3 (0.7-2.0) | 0.9 (0.3-2.8) | 0.7 (0.1-5.9) | 68 |
| Living on country |  |  |  |  |  |  |
| Does not currently live on country (Ref) |  |  |  |  |  | 332 |
| Currently living on country | 1.1 (0.6-2.0) | 0.4 (0.1-1.5) | 1.2 (0.6-2.4) | 1.9 (0.7-5.0) | 1.5 (0.3-8.1) | 74 |
| Has never lived on country (Ref) |  |  |  |  |  | 300 |
| Has lived on country | 1.1 (0.6-1.8) | **3.5 (1.0-12.5)*** | 1.2 (0.6-2.2) | 1.5 (0.5-4.5) | 2.4 (0.3-20.1) | 106 |
| History of family removals |  |  |  |  |  |  |
| Not part of Stolen Generations (Ref) |  |  |  |  |  | 395 |
| Part of Stolen Generations | 1.6 (0.4-5.9) | **-** | **-** | 5.6 (1.0-33.0) | **-** | 11 |
| Parents were not part of Stolen Generations (Ref) |  |  |  |  |  | 364 |
| Parents were part of Stolen Generations | 0.9 (0.4-2.0) | 0.7 (0.2-3.3) | 0.9 (0.4-2.3) | 2.0 (0.7-6.0) | **-** | 42 |
| Grandparents were not part of Stolen Generations (Ref) |  |  |  |  |  | 247 |
| Grandparents were not part of Stolen Generations | 0.8 (0.5-1.2) | 0.5 (0.2-1.2) | 1.2 (0.7-2.0) | 0.9 (0.4-2.1) | 1.5 (0.4-6.5) | 159 |
| Cultural experiences |  |  |  |  |  |  |
| Always/often proud to identify (Ref) |  |  |  |  |  | 394 |
| Sometimes/rarely/never proud to identify | **-** | 1.8 (0.4-8.9) | 1.7 (0.4-6.6) | 1.1 (0.1-9.1) | 3.3 (0.4-29.6) | 12 |
| Always/often feel a sense of connection and belonging (Ref) |  |  |  |  |  | 328 |
| Sometimes/rarely/never feel a sense of connection and belonging | 1.5 (0.9-2.7) | 2.1 (0.9-4.7) | 1.4 (0.7-2.6) | 0.7 (0.2-2.2) | 1.9 (0.4-8.2) | 78 |
| Always/often participates in community events (Ref) |  |  |  |  |  | 297 |
| Sometimes/rarely/never participates in community events | 1.2 (0.7-2.1) | **2.3 (1.1-5.0)*** | 1.2 (0.7-2.1) | 0.4 (0.1-1.3) | 1.2 (0.3-5.3) | 109 |
| Always/often feels empowered and strong to make positive choices (Ref) |  |  |  |  |  | 353 |
| Sometimes/rarely/never feels empowered and strong to make positive choices | **1.9 (1.0-3.7)*** | **3.2 (1.4-7.6)*** | 1.0 (0.5-2.1) | 0.5 (0.1-2.4) | 2.0 (0.4-10.6) | 53 |
| Sometimes experiences racism/discrimination (Ref) |  |  |  |  |  | 177 |
| Always/often experiences racism/discrimination | 0.8 (0.5-1.4) | 0.8 (0.3-2.0) | **0.4 (0.2-0.7)*** | **0.4 (0.3-1.0)*** | 3.1 (0.6-16.3) | 134 |
| Rarely/never experiences racism/discrimination | 0.9 (0.5-1.6) | 0.7 (0.3-2.0) | 0.5 (0.3-1.0) | 0.5 (0.2-0.7) | - | 95 |

*Reference groups are signified as (Ref)*

** Significance based on 95% Confidence Intervals (CI)*

Table 9. Sociodemographic and cultural correlates of lifetime mental disorders

|  | **Any mental disorder** | **Major depressive episode** | **Generalised anxiety disorder** | **Post-traumatic stress disorder** |  |
| --- | --- | --- | --- | --- | --- |
| **Sociodemographic variable** | **Odds Ratio (95% CI)** | | | | **N** |
| Age |  |  |  |  |  |
| 40+ (Ref) |  |  |  |  | **219** |
| 18-39 | 0.9 (0.6-1.4) | 0.9 (0.6-1.3) | 1.0 (0.6-1.8) | 1.3 (0.7-1.9) | 187 |
| Marital status |  |  |  |  |  |
| Married/De facto/Partnered (Ref) |  |  |  |  | 226 |
| Divorced/Separated/Widowed | 1.6 (0.7-3.9) | 0.7 (0.3-1.6) | 1.2 (0.8-2.6) | 1.7 (0.7-4.1) | 29 |
| Single/Other^a^ | 1.3 (0.8-2.0) | 0.8 (0.5-1.3) | 2.1 (0.8-5.9) | **1.7 (1.1-2.7)*** | 151 |
| Highest year of school completed |  |  |  |  |  |
| Year 12/equivalent (Ref) |  |  |  |  | 342 |
| Did not complete school^b^ | 1.1 (0.6-1.9) | 0.7 (0.4-1.2) | 1.0 (0.5-2.2) | 1.0 (0.6-1.9) | 64 |
| Highest tertiary qualification |  |  |  |  |  |
| Certificate/Diploma/Associate degree (Ref) |  |  |  |  | 189 |
| Bachelor/Post-graduate degree | 1.5 (0.8-2.6) | 1.3 (0.8-2.3) | 1.8 (0.9-3.5) | 0.7 (0.4-1.2) | 76 |
| No qualifications/Prefer not to say | 0.7 (0.5-1.2) | 1.3 (0.8-2.3) | 0.7 (0.3-1.3) | 0.7 (0.4-1.1) | 141 |
| Employment status |  |  |  |  |  |
| Paid employment (Ref) |  |  |  |  | 256 |
| Government payments | 1.2 (0.7-2.0) | 0.9 (0.5-1.6) | 1.6 (0.8-3.1) | 1.4 (0.8-2.5) | 87 |
| Unemployed/Studying without source of income | 0.7 (0.4-1.3) | 0.9 (0.5-1.6) | 1.1 (0.5-2.3) | 0.7 (0.4-1.4) | 63 |
| Living situation |  |  |  |  |  |
| Renting (Ref) |  |  |  |  | 235 |
| Homeowner | 1.0 (0.6-1.6) | 1.0 (0.6-1.6) | 1.0 (0.5-1.9) | 0.9 (0.6-1.5) | 118 |
| Staying with friends/family | 0.7 (0.4-1.4) | 0.7 (0.2-3.0) | 0.9 (0.4-2.2) | 1.3 (0.6-2.5) | 44 |
| Sleeping rough/Homeless/ Other | 1.2 (0.3-5.1) | 1.0 (0.6-1.6) | 1.4 (0.6-7.1) | 1.9 (0.5-7.8) | 9 |
| Financial stress (in last 12 months) |  |  |  |  |  |
| Able to pay bills (Ref) |  |  |  |  | 309 |
| Unable to pay bills | **1.9 (1.1-3.2)*** | **1.6 (1.1-2.7)*** | **2.0 (1.1-3.6)*** | 1.5 (0.9-2.5) | 97 |
| Able to afford groceries (Ref) |  |  |  |  | 328 |
| Unable to afford groceries | **2.0 (1.1-3.5)** | **2.0 (1.2-3.4)*** | 1.2 (0.6-2.3) | 1.5 (0.8-2.5) | 78 |
| Did not seek assistance from welfare (Ref) |  |  |  |  | 297 |
| Sought assistance from welfare | 1.6 (1.0-2.7) | 1.4 (0.9-2.3) | 0.9 (0.5-1.7) | **2.1 (1.3-3.4)*** | 109 |
| Did not seek financial help from friends/family (Ref) |  |  |  |  | 266 |
| Sought financial help from friends/family | **2.0 (1.3-2.3)*** | **1.6 (1.0-2.4)*** | 1.7 (0.9-3.0) | **1.9 (1.2-3.0)*** | 140 |
| History of incarceration |  |  |  |  |  |
| No incarceration (Ref) |  |  |  |  | 380 |
| Incarceration (youth and/or adult) | 0.9 (0.4-2.2) | 0.4 (0.1-1.1) | 1.8 (0.7-5.0) | 2.2 (0.9-5.2) | 26 |
|  |  |  |  |  |  |
|  | **Any mental disorder** | **Major depressive episode** | **Generalised anxiety disorder** | **Post-traumatic stress disorder** |  |
| **Cultural variable** | **Odds Ratio (95% CI)** | | | | **N** |
| Identification of Mob |  |  |  |  |  |
| Mob identified (Ref) |  |  |  |  | 363 |
| No Mob identified – don’t know/unsure | 1.1 (0.6-2.3) | 1.8 (0.9-3.6) | 1.5 (0.7-3.4) | **2.2 (1.1-4.2)*** | 43 |
| Identification of Country |  |  |  |  |  |
| Considers a place to be Country (Ref) |  |  |  |  | 338 |
| Does not consider a place to be Country/not sure | 0.9 (0.5-1.6) | 0.1 (0.6-1.7) | 0.4 (0.2-1.1) | 1.3 (0.8-2.4) | 68 |
| Living on country |  |  |  |  |  |
| Does not currently live on country (Ref) |  |  |  |  | 332 |
| Currently living on country | 1.2 (0.7-2.1) | 1.1 (0.6-1.8) | **0.3 (0.1-0.7)*** | 1.1 (0.6-1.9) | 74 |
| Has never lived on country (Ref) |  |  |  |  | 300 |
| Has lived on country | 1.5 (0.9-2.4) | 1.3 (0.8-2.1) | 1.7 (0.8-3.4) | 1.1 (0.7-1.8) | 106 |
| History of family removals |  |  |  |  |  |
| Not part of Stolen Generations (Ref) |  |  |  |  | 395 |
| Part of Stolen Generations | 0.6 (0.2-2.4) | 1.3 (0.3-4.8) | 0.7 (0.1-5.3) | **0.1 (0.0-0.7)*** | 11 |
| Parents were not part of Stolen Generations (Ref) |  |  |  |  | 364 |
| Parents were part of Stolen Generations | 1.4 (0.7-2.9) | 1.4 (0.7-2.7) | 0.5 (0.2-1.5) | 1.5 (0.7-2.9) | 42 |
| Grandparents were not part of Stolen Generations (Ref) |  |  |  |  | 247 |
| Grandparents were not part of Stolen Generations | 0.9 (0.6-1.4) | 1.0 (0.7-1.6) | 1.1 (0.6-1.9) | 1.5 (0.7-2.9) | 159 |
| Cultural experiences |  |  |  |  |  |
| Always/often proud to identify (Ref) |  |  |  |  | 394 |
| Sometimes/rarely/never proud to identify | 2.5 (0.5-12.2) | 0.7 (0.2-2.3) | 0.8 (0.2-3.7) | 1.6 (0.5-5.3) | 12 |
| Always/often feel a sense of connection and belonging (Ref) |  |  |  |  | 328 |
| Sometimes/rarely/never feel a sense of connection and belonging | **2.3 (1.3-4.1)*** | 1.5 (0.9-2.5) | 1.4 (0.7-2.6) | **2.1 (1.3-3.6)*** | 78 |
| Always/often participates in community events (Ref) |  |  |  |  | 297 |
| Sometimes/rarely/never participates in community events | **2.4 (1.4-4.1)*** | 1.6 (1.0-2.5) | **1.9 (1.1-3.4)*** | **1.9 (1.2-3.1)*** | 109 |
| Always/often feels empowered and strong to make positive choices (Ref) |  |  |  |  | 353 |
| Sometimes/rarely/never feels empowered and strong to make positive choices | **2.1 (1.1-4.2)*** | 1.4 (0.7-2.5) | 1.6 (0.8-3.4) | 1.7 (0.9-3.2) | 53 |
| Sometimes experiences racism/discrimination (Ref) |  |  |  |  | 177 |
| Always/often experiences racism/discrimination | **0.4 (0.3-0.7)*** | **0.5 (0.3-0.8)*** | 0.7 (0.4-1.3) | **0.4 (0.3-0.7)*** | 134 |
| Rarely/never experiences racism/discrimination | 0.6 (0.3-1.0) | 0.7 (0.4-1.3) | 0.7 (0.3-1.4) | **0.6 (0.3-1.0)*** | 95 |

*Reference groups are signified as (Ref)*

** Significance based on 95% Confidence Intervals (CI)*
